# Editorial: The Impact of Microorganisms on Consumption of Atmospheric Trace Gases

**DOI:** 10.3389/fmicb.2017.01856

**Published:** 2017-09-28

**Authors:** Steffen Kolb, Marcus A. Horn, J. Colin Murrell, Claudia Knief

**Affiliations:** ^1^Leibniz Zentrum für Agrarlandschaftsforschung e.V., Institute Landschaftsbiogeochemie, Müncheberg, Germany; ^2^Bodenmikrobiologie, Institut für Mikrobiologie, Leibniz-Universität Hannover, Hannover, Germany; ^3^School of Environmental Sciences, University of East Anglia, Norwich, United Kingdom; ^4^Molekularbiologie der Rhizosphäre, Institut für Nutzpflanzenwissenschaften und Ressourcenschutz, Rheinische Friedrich-Wilhelms-Universität Bonn, Bonn, Germany

**Keywords:** methane, methanotroph, nitrous oxide, denitrification, volatile organic compounds, carbon monoxide, phyllosphere

Gases with a mixing ratio of <1% in the atmosphere are considered as trace gases. Several of these trace gases originate from biological processes in marine and terrestrial ecosystems and are of relevance for the climate as they contribute to global warming, to the troposphere's chemical reactive system that builds the ozone layer, or they impact on the stability of aerosols, greenhouse, and pollutant gases (Conrad, 2009; Arneth et al., 2010; Penuelas and Staudt, 2010). These gases include methane (CH_4_), a multitude of volatile organic compounds of biogenic origin (bVOCs) and inorganic gases such as nitrogen oxides or ozone (Conrad, 2009; Hewitt et al., 2011; Peñuelas et al., 2014; Fowler et al., 2016). The important role of microorganisms for trace gas cycling has been intensively studied for the greenhouse gases nitrous oxide (N_2_O) and methane (CH_4_), but is less well-understood for organisms that metabolize H_2_, carbon monoxide (CO), or bVOCs. The studies compiled in this *Research Topic* reflect this very well. While a number of articles focus on N_2_O and CH_4_ cycling or CO oxidation, only a few articles address conversion processes of bVOCs (Dixon et al.; Nadalig et al.). The *Research Topic* is complemented by three review articles about the consumption of CH_4_ and monoterpenes, as well as the role of the phyllosphere as a particular habitat for trace gas-consuming microorganisms, and points out future research directions in the field (Marmulla and Harder; Bringel and Couee; Knief).

Articles in this research topic on N_2_O cycling are related to terrestrial environments and denitrification. Soil N_2_O emissions result from different biological processes with nitrification and denitrification being the most important ones. Denitrifiers are facultative aerobic microorganisms that reduce nitrate (${\text{NO}}_{3}^{-}$) or nitrite (${\text{NO}}_{2}^{-}$) to the gaseous products nitric oxide (NO), nitrous oxide (N_2_O), or dinitrogen (N_2_) under oxygen-limited conditions. Various environmental factors have been identified that affect the composition, abundance, and activity of these microbial groups (reviewed in Braker and Conrad, 2011). The study of Brenzinger et al. evaluates changes on the denitrifier microbiota and gene expression upon pH shifts. Their observation of changes in gene expression of specific taxa demonstrates that functional redundancy of the soil microbiome is important to maintain denitrification upon acidification. Functional redundancy and process partitioning among ${\text{NO}}_{3}^{-}$- and N_2_O-reducing denitrifiers can also explain the findings that *in situ* N_2_O fluxes were largely unaffected upon manipulations of the water table in a wetland, although an increase in N_2_O reduction activity was observed upon flooding (Palmer et al.). In contrast, land-use change of an agroforest system to an open area converted a cork oak wood from a N_2_O source to a sink (Shvaleva et al.). This change was not reflected in the abundance of the denitrifier gene *nosZ*. All of these studies demonstrate that the underlying microbial processes that control N_2_O fluxes are complex and require a comprehensive analysis of all the different microbial groups that may be involved. Moreover, information about microbiome composition needs to be complemented by data on taxon abundance, activity, and ecophysiology of isolates in order to obtain an understanding of the mechanisms underpinning the observable net ecosystem N_2_O fluxes.

To better assess the biotic mechanisms driving N_2_O fluxes, *in vitro* analyses of pure cultures are helpful, as exemplified by two studies in this *Research topic*. Bueno et al. revealed that the well-known soil microorganism *Ensifer meliloti* strain 1021 displays a phenotype that enables it to function as a sink for N_2_O due to anaerobic N_2_O respiration. Remarkably, it appears that this strain is not able to grow *via* ${\text{NO}}_{3}^{-}$ respiration, although it can express all genes encoding a complete set of denitrification enzymes. More work is needed to elucidate this peculiarity. Kits et al. studied denitrification activity in the methanotroph *Methylomicrobium album* BG8. Although methanotrophs are considered to be the major biological sink for CH_4_ in soil, some of them also form N_2_O through denitrification under hypoxic conditions. The authors prove in their study that *M. album* BG8 carries out denitrification using nitrite as electron acceptor while oxidizing a range of different one- and even two-carbon compounds, i.e., ethane and ethanol.

The microbial ecology of methanotrophs has been studied for decades, due to the importance of CH_4_ as greenhouse gas. This has substantially increased knowledge on their ecophysiology and environmental distribution, as summarized in the review by Knief. Methanotrophic communities are often analyzed based on the marker gene *pmoA*, encoding a subunit of the membrane-bound methane monooxygenase. The extensive sequencing of *pmoA* has led to the identification of a number of new taxa. Based on this information, the occurrence of the different taxa in diverse ecosystems was analyzed within a meta-analysis, thus providing new knowledge about habitat preferences of specific methanotroph taxa. Some methanotroph taxa were identified as specialists, occurring only in specific ecosystems, while other genera appear to be generalists (Knief). Rather specific habitat preferences have for example been suggested for a group of uncultured atmospheric CH_4_-consuming methanotrophs (termed USCα), with upland soils and especially forest soils being the preferred habitat (Kolb, 2009). The conversion of an Amazonian forest into a manioc (cassava) plantation resulted in drastically decreased abundance of this group of methanotrophs concomitant with the CH_4_ sink activity (Lima et al.). Remarkably, this response was only observed in one of the two soils studied. In an Amazonian Dark Earth (syn. terra preta) soil, USCα was still abundant and CH_4_ uptake rates remained high 5 years after deforestation and manioc cultivation. The causes for this difference are unclear and need further investigation.

Research on the biological cycling of other one-carbon VOCs such as CO or chloromethane has attracted less attention until now, despite the fact that these gases play a crucial role in atmospheric chemistry and impact on the global climate (Daniel and Solomon, 1998; Harper, 2000). The *Research Topic* includes three studies addressing CO oxidation, which demonstrate nicely the different experimental approaches that can be taken to study a specific functional guild in the environment. Lynch et al. studied trace gas consumers in dry high-elevation mineral soils originating from volcanic deposits in the Atacama Desert via a metagenomic analysis of the soil microbiome, and mined for genes and pathways known to be involved in trace gas conversion. The authors were able to reconstruct the genome of the dominant taxon, *Pseudonocardia* sp., and revealed genetic potential of this organism for hydrogen (H_2_), CO, and some further one-carbon compounds oxidation. In the study by Quiza et al., aerobic CO oxidizing microorganisms were specifically detected by targeting a functional gene marker, *coxL*, encoding the large subunit of the CO-dehydrogenase. Highest activities, along with the detection of distinct aerobic CO oxidizers, were observed in a deciduous forest soil, compared to an adjacent afforested larch plantation and a maize field. Brady et al. applied stable isotope probing (SIP) in combination with 16S rRNA gene sequencing. SIP is a valuable method to specifically identify the metabolically active microbiome members but requires ^13^CO_2_ controls to distinguish between CO- and CO_2_-assimilating microorganisms. Brady et al. identified novel members of *Firmicutes* as autotrophic CO consumers in oxygen-limited geothermal springs using this experimental approach. The fact that heterotrophic carboxydotrophs, which use CO only as an energy source, will be missed by SIP highlights the need for the application of complementary approaches to comprehensively assess the role of CO consumers in the environment.

Chloromethane is a bVOC, and the most abundant halogenated compound in the atmosphere. The only known aerobic pathway for chloromethane oxidation is the *cmu* pathway, which has been well-investigated in aerobic *Alphaproteobacteria* (Nadalig et al.). Based on a genomic survey, Nadalig et al. show that the *cmu* pathway occurs in several other bacterial taxa, including obligate anaerobes. Interestingly, the authors found that the genome of the aerobe *Leisingeria methylhalidivorans*, i.e., a known chloromethane degrader, does not have the *cmu* pathway. Moreover, the authors proved that *L. methylhalidivorans* has diverging isotopic signatures of ^13^C and ^2^H when assimilating chloromethane compared to other alphaproteobacterial *cmu*-containing strains, suggesting the existence of a hitherto unknown pathway. This study demonstrates the limited knowledge we currently have about microbial bVOC metabolism. Another example is given by the review of Marmulla and Harder who provide an overview on the complex biochemistry and pathways of monoterpene degradation in microorganisms and conclude that these pathways need to be studied in more detail in the future. Considering the multitude of monoterpenes present in nature, it is evident that more research is needed. Knowledge about the biochemistry, the genetic makeup of the metabolic pathways, and their distribution among microorganisms is an indispensable prerequisite to perform environmental studies with the aim to improve our understanding of how microbiomes control trace gas fluxes.

The aforementioned studies of the *Research Topic* highlight that our current knowledge about the identity of microorganisms involved in the production or consumption of trace gases and, in particular bVOCs, is limited. For most bVOCs, the impact of microbial consumption on net fluxes between soil or marine ecosystems and the atmosphere is not clear to date. The study of Dixon et al. is one rare example on microbial acetone oxidation in marine waters with seasonal resolution and estimates the quantitative proportion of acetone carbon consumption compared to other bVOCs. Remarkably, the data suggest the existence of an unrecognized production mechanism for acetone during the winter. Besides soils and aquatic habitats, another major habitat for trace gas metabolizing microorganisms is the plant leaf surface, often referred to as the phyllosphere. Considering that plants are major producers of bVOCs (Penuelas and Staudt, 2010), their leaves represent an important interface to the atmosphere and are colonized by microorganisms. Bringel and Couee emphasize in their review the need to study this aspect in more detail. The authors summarize current knowledge about phyllosphere microorganisms metabolizing bVOCs, which is largely limited to one-carbon compounds.

Knowledge about microbial consumption of bVOCs is far from being compared to knowledge on microbial consumption of CH_4_ or N_2_O. This includes the quantitative relevance of microbial activities at the ecosystem level and globally, the identities of trace gas degrading microorganisms, the analysis of their degradation pathways, as well as the physiological traits that finally determining the activity of these microbes *in situ*. Thus, the research field of microbial trace gas consumption can be considered to be still in its infancy and necessitates future research to better quantitatively consider microbial trace gas conversions at an ecosystem level, e.g., in upcoming modeling efforts (Graham et al., 2016).

## Author contributions

All authors were involved in the set-up and design of this Research Topic. SK and CK wrote this editorial, MH and JM provided valuable comments.

### Conflict of interest statement

The authors declare that the research was conducted in the absence of any commercial or financial relationships that could be construed as a potential conflict of interest.
